# Approach to Diagnosis and Management of Polycystic Ovary Syndrome in Bangladesh: A Nationwide Cross-Sectional Survey of Physicians

**DOI:** 10.7759/cureus.93912

**Published:** 2025-10-06

**Authors:** Marufa Mustari, Ahmed Ifrad Bin Raunak, Samira Mahjabeen, Syed Azmal Mahmood, Faria Afsana, M Saifuddin, Tahniyah Haq, Sunjida Islam, Tanjina Hossain, S M Mohiuddin, Nazma Akter, Afsar Ahammed, Rezwana Sobhan, Shahin Ibn Rahman, Sourav Sarkar, Md. Faruque Pathan, Muhammad Hafizur Rahman, Afia Zainab Tanni, Shahjada Selim

**Affiliations:** 1 Department of Endocrinology, Bangabandhu Sheikh Mujib Medical University, Dhaka, BGD; 2 Department of Endocrinology, Mugda Medical College Hospital, Dhaka, BGD; 3 Department of Endocrinology, Dhaka Medical College Hospital, Dhaka, BGD; 4 Department of Endocrinology, BIRDEM (Bangladesh Institute of Research and Rehabilitation in Diabetes, Endocrine and Metabolic Disorders) General Hospital, Dhaka, BGD; 5 Department of Endocrinology, Dhaka Medical College, Dhaka, BGD; 6 Department of Endocrinology, Eastern Medical College and Hospital, Cumilla, BGD; 7 Department of Endocrinology and Metabolism, Green Life Medical College, Dhaka, BGD; 8 Department of Endocrinology, Sir Salimullah Medical College, Dhaka, BGD; 9 Department of Endocrinology, MARKS Medical College &amp; Hospital, Dhaka, BGD; 10 Department of Endocrinology, National Institute of Traumatology and Orthopaedic Rehabilitation, Dhaka, BGD; 11 Department of Endocrinology, Enam Medical College Hospital, Dhaka, BGD; 12 Department of Medicine, Boalkhali Upazila Health Complex, Chattogram, BGD; 13 Department of Endocrinology, BIRDEM (Bangladesh Institute of Research and Rehabilitation in Diabetes, Endocrine and Metabolic Disorders) Academy, Dhaka, BGD; 14 Department of Endocrinology, United Hospital, Dhaka, BGD; 15 Department of Endocrinology, National Institute of Burn &amp; Plastic Surgery, Dhaka, BGD

**Keywords:** endocrinologists, gynecologists, polycystic ovary syndrome, rotterdam criteria, screening and management guidelines

## Abstract

Background: Polycystic ovary syndrome (PCOS) is the most prevalent endocrine disorder among women of reproductive age. This nationwide cross-sectional study aimed to assess physicians' diagnostic and management practices for PCOS in Bangladesh, with a particular focus on adherence to established guidelines.

Methods: This nationwide cross-sectional survey was conducted between April and October 2024 among general practitioners and specialists using a semi-structured questionnaire. Information on demographic characteristics, diagnostic and therapeutic practice patterns, and use of clinical guidelines was collected. Descriptive statistical methods were applied.

Results: Among the 643 practicing physicians, 553 (86%) were specialists in Gynecology and Obstetrics, followed by general practitioners (7.8%), endocrinologists (4%), and others. The European Society for Human Reproduction and Embryology (ESHRE) was the most commonly followed guideline (37%), along with other evidence-based guidelines. Infertility, irregular menstrual cycles, and hirsutism were frequent presenting symptoms, while infertility, type 2 diabetes, and obesity were identified as major long-term risks. Lifestyle modifications (515, 80%) and metformin (466, 72.5%) were the most common treatment strategies. Overall, 573 (89.1%, 95% CI: 86.7%-91.5%) physicians reported applying at least one recognized guideline. However, advising follow-up varied, with only 180 (52%) recommending follow-ups within 12 weeks. Furthermore, 465 (74.6%) participants who wanted a referral system supported the establishment of a structured referral system, with endocrinologists and gynecologists as the preferred specialists.

Conclusion:While most physicians reported familiarity with international guidelines, variations in adherence and follow-up practices were observed. The development of a unified, context-specific national guideline is recommended to strengthen standardization of care and improve patient outcomes in Bangladesh.

## Introduction

Polycystic ovary syndrome (PCOS) is a complex endocrine disorder characterized by menstrual irregularities, hyperandrogenism, and the presence of ovarian cysts. It is associated with serious comorbidities, including infertility, obesity, type 2 diabetes, cardiovascular disease, and psychiatric disorders such as anxiety and depression [1,2]. The disease has a significant public health impact affecting women of reproductive age, with an estimated global prevalence of approximately 16 million individuals [3,4]. In Bangladesh, the reported prevalence of PCOS varies widely, ranging from 6.11% among women attending gynecology outpatient departments to 92.16% among those consulting for hirsutism [5].

Over the past two decades, substantial progress has been made in understanding the epidemiology, phenotypes, evaluation, management, genetics, and long-term complications of PCOS. Various medical societies, including the National Institutes of Health (NIH), the Androgen Excess-PCOS (AE-PCOS) Society, and the Rotterdam consensus group, have published guidelines to aid in the diagnosis, treatment, and management of PCOS [6,7]. While these guidelines emphasize reproductive health aspects, PCOS impacts metabolic health and psychological well-being, necessitating a holistic approach to care. Diagnosing PCOS remains challenging due to multiple diagnostic criteria, inconsistent definitions of clinical and biochemical hyperandrogenism, varying phenotypes, and differences in presentation by age and ethnicity. Management typically focuses on symptom relief and prevention of long-term complications [8]. Early diagnosis is critical for regulating the menstrual cycle, controlling hyperandrogenism, and mitigating insulin resistance to reduce the risk of type 2 diabetes and cardiovascular disease. Early intervention also supports lifestyle changes and medical management to address obesity, improve fertility, alleviate psychological distress, and enhance overall quality of life [2,9]. Despite the availability of international guidelines, there is often a gap between evidence-based recommendations and clinical practice [10-12].

Globally, suboptimal diagnostic experiences and insufficient information provision for PCOS highlight the need for improved care [13,14]. A 2017 survey in the USrevealed that only 41% of practicing obstetricians and gynecologists and 68% of reproductive endocrinologists and infertility specialists were aware of the Rotterdam criteria for diagnosing PCOS [3]. In Europe, approximately three-quarters of obstetrician-gynecologists and endocrinologists use the Rotterdam criteria, while in North America, roughly half prefer the NIH criteria [12]. A recent international survey of gynecology and reproductive endocrinology practitioners reported similar findings [15].

In Bangladesh, PCOS is increasingly prevalent among adolescents and women of reproductive age [16], although approximately 64% of symptomatic women remain undiagnosed [17]. In the absence of a standardized referral system, patients often seek clinical consultations from diverse specialties and centers such as medical college hospitals, local hospitals, or even local drug vendors [18,19]. The absence of coordinated care pathways means that patients often receive fragmented care. For instance, women with acne or hirsutism might initially consult dermatologists, who may incidentally diagnose PCOS. Those experiencing menstrual irregularities commonly consult gynecologists or obstetricians. Patients with metabolic abnormalities, such as insulin resistance, might be diagnosed by endocrinologists or even cardiologists. This lack of streamlined referral processes can result in inconsistent diagnoses and hinder timely, comprehensive management. Moreover, the diversity of specialists involved, often without coordinated communication, can lead to suboptimal care for these patients.

As multiple international guidelines exist, notable differences are seen in diagnostic methods, knowledge, and treatment practices between different specialists. In a recent meta-analysis, the prevalence of PCOS in adolescents based on the Rotterdam criteria was 11.04%; based on the NIH criteria, it was 3.39%; and based on the Androgen Excess and Polycystic Ovary Syndrome Society, it was 8.03% [20]. However, due to the absence of a referral system and specific strategies regarding the management of PCOS in a low-resource setting like Bangladesh, there is a dearth of information available regarding the adherence to guidelines among physicians in Bangladesh. This limits understanding of local diagnostic patterns, adherence to international guidelines, and referral practices, which are essential for adapting context-specific interventions. This study aims to investigate the diagnostic and management practices for PCOS among physicians in Bangladesh. Understanding current practices is essential for identifying areas for improvement and implementing evidence-based guidelines effectively in clinical settings.

## Materials and methods

Study design and participant selection

This nationwide cross-sectional study was conducted among general practitioners and specialists working in public and private hospitals across Bangladesh. Physicians were contacted through the professional networks of the principal investigator and her team, with efforts to include participants from all divisions. The study period was between April 2024 and October 2024. Physicians from various specialties were included along with the general practitioners.The study includes specialists from the following disciplines: 1) Endocrinology, 2) Gynecology & Obstetrics, 3) Internal Medicine, 4) Cardiology, 5) Psychiatry, 6) Dermatology, and 7) Surgery.

Sample size estimation

The sample size was calculated using the formula for a 95% confidence level (z = 1.96), with an assumed prevalence of 0.5 (p) and a margin of error of 0.05 (d). Given the study's multicenter design, a design effect of 1.5, a 90% response rate, and a 10% non-response rate were factored in, resulting in a required sample size of 698 participants. This study employed a convenience purposive sampling method, including only registered physicians actively involved in patient care. Recruitment was initiated through the investigators' networks, with participants encouraged to share the questionnaire with their colleagues. After excluding 55 incomplete responses, 643 physicians were included in the final analysis.

Study procedure

A semi-structured questionnaire was designed to collect information in two main sections: Sociodemographic characteristics: Specialty, years of clinical experience, practice setting, and patient volume. PCOS-specific practices: Number of PCOS patients treated weekly, common clinical presentations, diagnostic approaches, treatment strategies, follow-up schedules, adherence to clinical guidelines, referral practices, and long-term management concerns.

The questionnaire comprised 23 questions and was designed to be completed within 10 to 20 minutes. It was developed through an extensive literature review and consultations with academic faculty. The questionnaire, provided in English, included both open-ended and closed-ended questions. A pilot test involving 10 physicians from Bangabandhu Sheikh Mujib Medical University (BSMMU) was conducted to ensure clarity and reliability. Participation was voluntary, with responses anonymized to protect confidentiality. Participants were instructed to complete the survey only once, and no question was mandatory. Further details are provided in the Appendix.

Ethical considerations

Ethical approval was obtained from the Institutional Review Board (IRB) of Bangabandhu Sheikh Mujib Medical University, Dhaka, Bangladesh, and written informed consent was secured from all participants. The study adhered to the ethical principles outlined in the Declaration of Helsinki and its subsequent amendments.

Statistical analysis

Following data collection, the dataset was cleaned and organized using Microsoft Excel (Microsoft, Redmond, WA). Statistical analysis was performed using SPSS version 26.0 (IBM Corp., Armonk, NY). Summary statistics were employed, with categorical variables presented as frequencies and percentages and continuous variables as means with standard deviations (SDs).

## Results

Physician characteristics and practice information

A total of 643 physicians participated voluntarily, recruited through professional networks. Some specific demographic details, like age and gender of the physicians, were excluded to focus on clinical practices regarding PCOS. The study aimed to identify physician specialty groups involved in PCOS care, assess their diagnostic and treatment approaches, and explore referral practices.

Among the participating physicians, 46% were from Dhaka, with the rest practicing across other cities and towns. Most were specialists in Gynecology and Obstetrics, followed by other specialties and physicians as general practitioners. Over half of these participants had more than 10 years of experience, and a majority (41.7%) of physicians managed less than 50 patients weekly. Among the physicians, only 10.9% managed PCOS patients during a week, where the majority (59.6%) had less than 10 PCOS patients per week. A majority of these physicians followed clinical guidelines, mainly the European Society of Human Reproduction and Embryology (ESHRE), but 10.9% were unsure which guidelines to use, revealing potential gaps in knowledge or access to standard care protocols. About 89.1% (95% CI: 86.7%-91.5%) of physicians followed clinical guidelines, mainly the ESHRE, where almost 10.9% were unsure which guidelines to use (Table 1). However, confidence intervals were not mentioned in the table.

**Table 1:** Baseline characteristics of the physicians (n = 643) Values are represented in frequency and percentage. PCOS, polycystic ovary syndrome.

Characteristics	n	%
Location of practice		
Inside Dhaka city	295	45.9
Outside Dhaka city	294	45.7
Not mentioned	54	8.4
Current discipline of practitioners		
Endocrinology	26	4
Internal medicine	6	0.9
Gynecology & Obstetrics	553	86
Cardiology	1	0.2
Dermatology	8	1.2
Surgery	1	0.2
General practitioners	50	7.8
Experience of clinical practice (years)		
<5	130	20.5
5-10	152	24.0
10-15	177	27.9
>15	175	27.6
Weekly number of total patients consulted		
<50	268	41.7
50-100	100	16.7
100-500	223	34.7
500-1000	6	0.9
>1000	3	0.5
Weekly number of PCOS patients consulted		
<10	383	59.6
10-20	183	28.5
>20	70	10.9
Physicians who prefer guidelines of PCOS	573	89.1
Physicians with no preferred guideline	70	10.9

Preferred guidelines for PCOS

**Figure 1:**
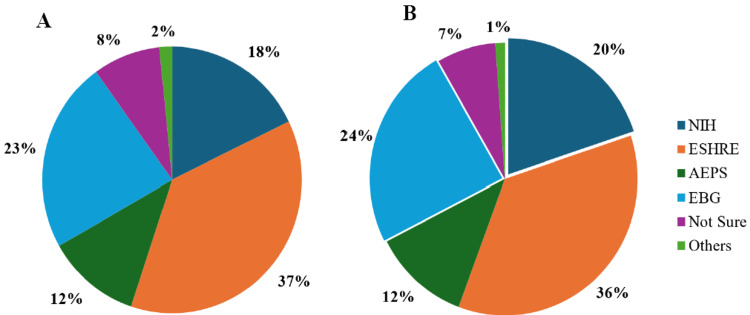
A. Preferred guidelines for screening PCOS; B. preferred guidelines for PCOS management PCOS, polycystic ovary syndrome; NIH, National Institutes of Health; ESHRE, European Society for Human Reproduction and Embryology; AEPS, Androgen Excess and PCOS Society; EBG,Evidence-Based Guidelines.

A majority of the participating physicians reported adherence to the Rotterdam/ESHRE2023 guidelines for both the screening and management of PCOS, followed by other known guidelines. However, about 7% to 9% of participants did not follow any specific guidelines for PCOS screening or management. Furthermore, a small proportion of physicians (1.7% for screening and 1.2% for management) reported relying on other recommendations for PCOS screening and treatment (Figure 1). These findings highlight variability in the application of guidelines, emphasizing the need for uniformity in clinical practice to ensure standardized patient care.

Guideline usage by specialty

**Figure 2:**
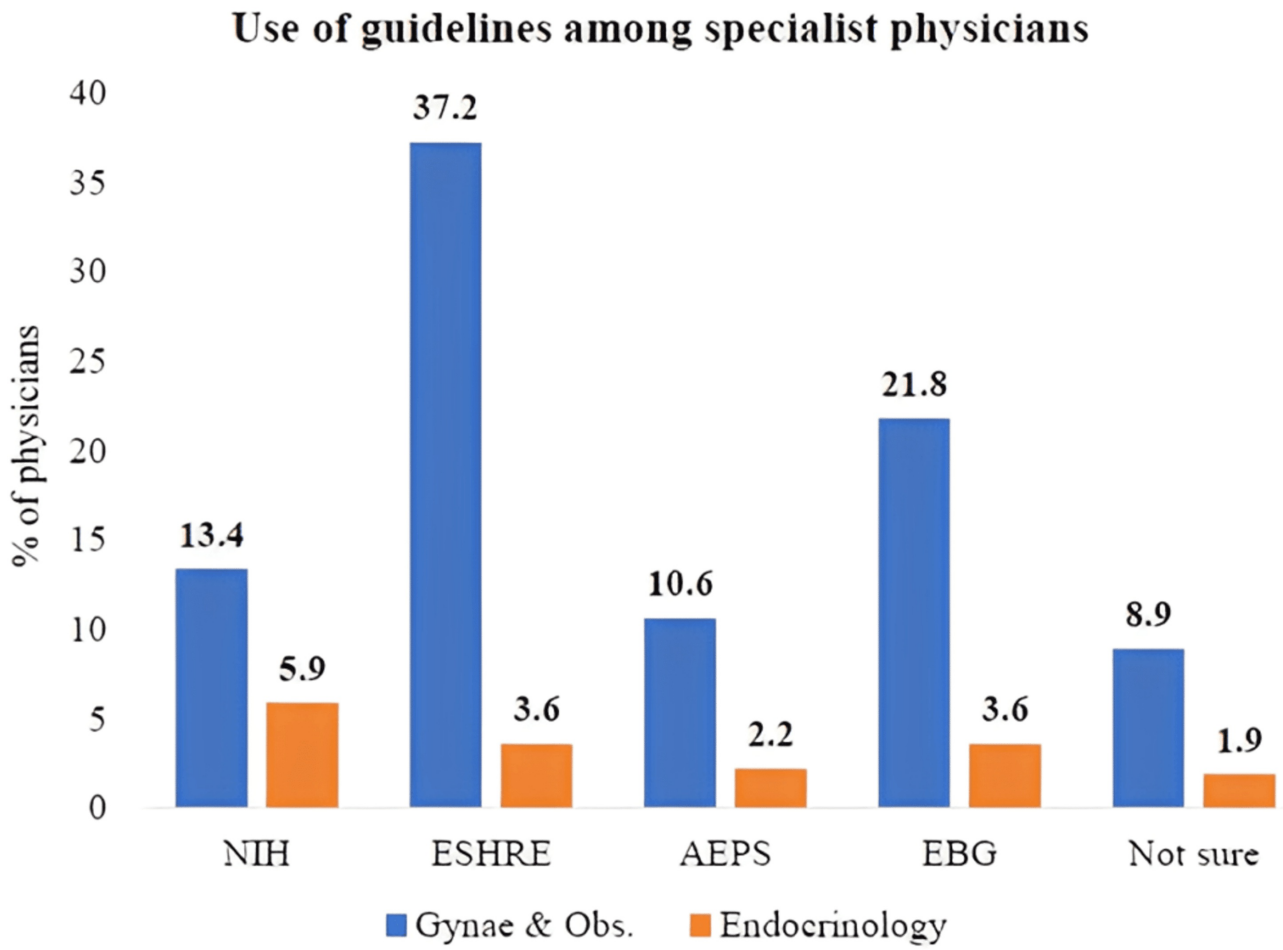
Comparison of the use of guidelines among gynecologists, endocrinologists, and other specialists (n = 643) PCOS, polycystic ovary syndrome; NIH, National Institutes of Health; ESHRE, European Society for Human Reproduction and Embryology; AEPS, Androgen Excess and PCOS Society; EBG,Evidence-Based Guidelines.

Figure 2 illustrates the distribution of guideline adherence among physicians managing PCOS, with percentages categorized by medical specialty. The ESHRE guidelines emerged as the most widely followed among Gynecology & Obstetrics physicians, whereas Evidence-Based Guidelines (EBG) were adhered to mostly by endocrinologists.

Diagnostic approaches for PCOS by physicians

In this study, most physicians diagnosed PCOS in patients presenting with menstrual disturbances, infertility, hirsutism, and obesity. For initial diagnosis and treatment, they frequently recommended abdominal ultrasound (76%), thyroid-stimulating hormone (TSH) testing (66.7%), serum luteinizing hormone (LH) (49.1%), and oral glucose tolerance tests (OGTT) (44.4%). A majority (65.6%) prioritized optimizing treatment, followed by counseling on lifestyle modifications and regular follow-ups, reflecting a comprehensive approach that addresses both reproductive and metabolic health(Table 2).

**Table 2:** Common clinical findings and diagnostic and management approaches preferred by physicians for PCOS patients (n = 643) Data are presented as n (%), unless stated otherwise. FSH, follicle-stimulating hormone; LH, luteinizing hormone; TSH, thyroid-stimulating hormone; DHEA, dehydroepiandrosterone; OGTT, oral glucose tolerance test; AMH,anti-Müllerian hormone; USG, ultrasonography. *Multiple responses were considered.

Variables	n	%
Common reasons for the patients attending the clinic*		
Hirsutism	327	49.3
Obesity	284	44.2
Menstrual disturbance	524	81.5
Metabolic disorders	57	8.9
Lack of fertility	445	69.2
Common laboratory tests prescribed*		
Serum FSH	184	28.6
Serum LH	316	49.1
TSH	429	66.7
Prolactin	297	26.2
DHEA	70	10.9
Free testosterone	209	32.5
Fasting insulin	137	21.3
17(OH) progesterone	14	2.2
OGTT	292	44.4
Fasting lipid profile	118	18.4
AMH	154	24
USG of the abdomen	489	76
Serum cortisol	17	2.6
Management plan of physicians*		
Optimizing treatment	422	31
Counselling of the patients	351	25
Referred to respective specialist	85	6
Physicians feel that patients with PCOS should come for follow-up on the given date	394	28
Follow-up	138	10

Management of PCOS by physicians

**Figure 3:**
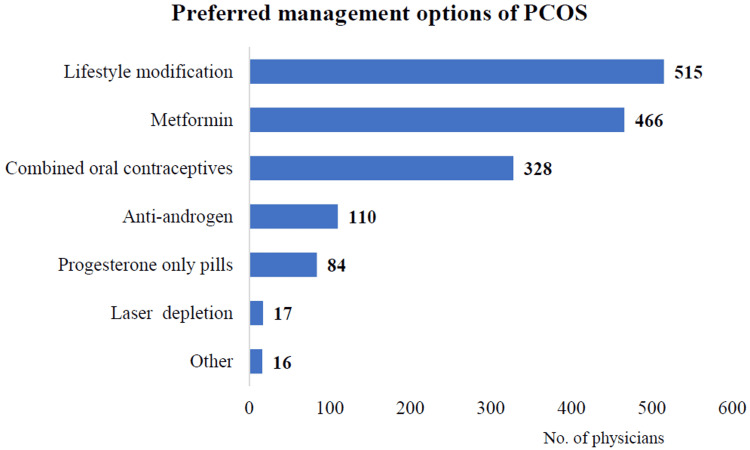
Management options preferred by physicians for PCOS patients (n = 643) PCOS, polycystic ovary syndrome.

Lifestyle modifications (80.1%, 95% CI: 77%-83.2%) were the most frequently recommended approach, followed by metformin, combined oral contraceptives, anti-androgens, and progesterone-only pills. Also, the less frequently chosen approaches include anti-androgens, progesterone-only pills, laser depletion for hair reduction, and other unspecified treatments. The data highlightlifestyle interventions and pharmacological strategies like metformin or contraceptives as primary management options for PCOS (Figure 3). However, the confidence interval is not shown in the figure.

**Figure 4:**
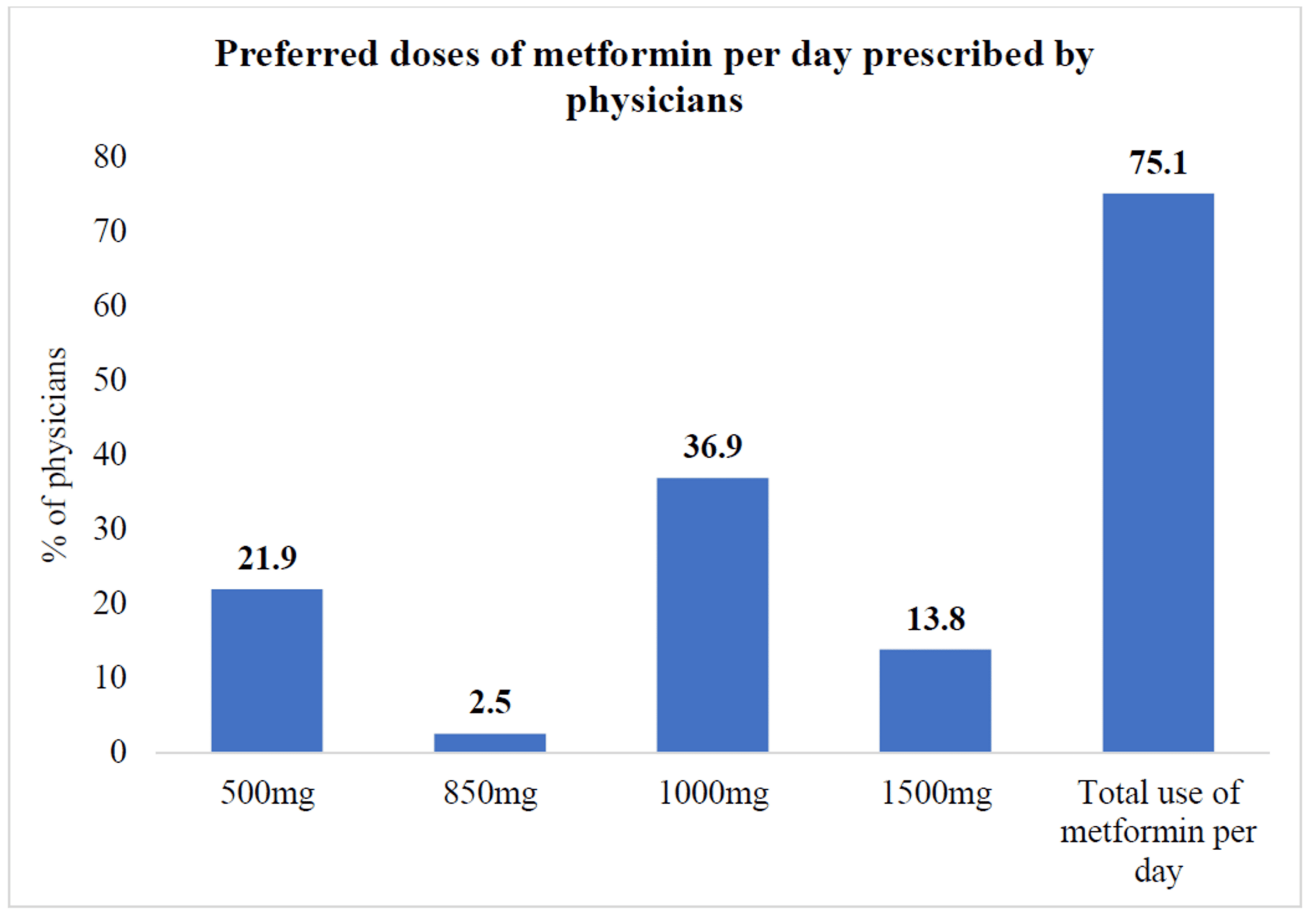
Dose of metformin per day preferred by physicians for management of PCOS (n = 643) PCOS, polycystic ovary syndrome.

Metformin appeared as the most commonly preferred treatment for PCOS, with 75.1% of physicians selecting it as their primary choice. The initial prescribed dose was typically 500 mg per day, gradually increasing based on patient response. The majority of physicians favored a daily dose of 1000 mg, administered in divided doses for optimal efficacy and tolerability. A higher dose of 1500 mg per day was less commonly prescribed, with only 13.8% of physicians choosing this regimen (Figure 4).

Follow-up of patients with PCOS

A majority of the participants reported scheduling follow-up visits for their PCOS patients, with 52% preferring a 12-week follow-up interval. The study also highlighted referral practices, with 74.6% of physicians referring patients to specialists, mainly endocrinologists, followed by gynecologists, dermatologists, and medicine specialists (Table 3).

**Table 3:** Preferred follow-up schedule, referral system, and long-term concerns of PCOS (n = 643) PCOS, polycystic ovary syndrome.

Variables	n	%
Preferred follow-up schedule of the physicians for PCOS patients		
In 4 weeks	29	9
In 6 weeks	29	9
In 8 weeks	17	5
In 12 weeks	180	52
In 24 weeks	58	17
Not sure	31	9
Physicians agreed to refer PCOS patients to specialists	465	74.6
Preferred specialists		
Endocrinologist	397	61.7
Gynecologist	203	31.6
Medicine specialist	7	1.1
Dermatologist	33	5.1
Others	2	0.4
Physicians’ long-term concerns about PCOS		
Type 2 diabetes	360	56
Infertility	566	88
Cardiovascular diseases	145	22.6
Obesity	358	55.7
Endometrial cancer	158	24.6
Psychological problems	172	26.7
Others	3	0.5

Infertility was the most common long-term concern for PCOS patients, mentioned by 88% of doctors. Other major concerns included diabetes (56%), obesity (55.7%), mental health issues (26.7%), uterine cancer (24.6%), and heart problems (22.6%). Only 0.5% of doctors mentioned concerns outside these areas.

In multivariate analysis, physicians practicing inside Dhaka were significantly more likely to adhere to PCOS guidelines (adjusted odds ratio [AOR] = 2.50, 95% CI: 1.20-5.21, p = 0.015). In contrast, those who preferred referring patients to specialists had lower odds of adherence (AOR = 0.31, 95% CI: 0.13-0.74, p = 0.009). No significant associations were observed for physician specialization, duration of experience, use of lifestyle modification, or follow-up perspectives (Table 4).

**Table 4:** Factors associated with physician adherence to different guidelines of PCOS using multivariate logistic regression PCOS, polycystic ovary syndrome.

Variables	Adjusted OR (95% CI)	p-Value
Category of specialization of physicians managingPCOS patients		
Endocrinologist	Ref	
Gynecology and Obstetrics	2.133 (0.847-5.369)	0.108
Duration of experiences		
<10 years	Ref	
≥10 years	1.267 (0.639-2.513)	0.498
Location of treatment of patients		
Outside Dhaka	Ref	
Inside Dhaka	2.495 (1.195-5.207)	0.015
Lifestyle modification as treatment		
No	Ref	
Yes	1.512 (0.679-3.367)	0.312
Preferred referral system to specialists of PCOS		
No	Ref	
Yes	0.310 (0.129-0.744)	0.009
Physician’s perspective on patient's follow-up visits		
Does not come to follow-ups	Ref	
Comes to follow-ups	1.557 (0.789-3.071)	0.201

## Discussion

The study provides critical insights into the practices of physicians in Bangladesh regarding the diagnosis and management of PCOS. A total of 643 physicians participated, with the majority specializing in gynecology and clustered in Dhaka. Most adhered to established guidelines, particularly the Rotterdam/ESHRE criteria for diagnosing PCOS. Physicians frequently encountered symptoms such as irregular menstrual cycles, infertility, and weight gain, relying on diagnostic tools like ultrasounds and hormonal assays. Nearly two-thirds of the management strategies emphasized lifestyle modifications, complemented by metformin and combined oral contraceptives, reflecting global best practices. However, gaps in knowledge and adherence to guidelines persist, as some physicians expressed uncertainty about which guidelines to follow. Long-term concerns, including infertility, metabolic disorders, and cardiovascular risks, underscore the need for comprehensive, evidence-based care and an effective referral system [10,11].

This study included physicians from diverse regions of the country, revealing that most followed at least one established guideline for PCOS management. The most commonly cited guidelines were those from the NIH, the Rotterdam/ESHRE criteria, the Endocrine Society, and the AE-PCOS Society. Gynecologists and obstetricians predominantly followed the ESHRE guidelines, while other specialists varied in their preferences. Dokras et al. reported that similar patterns have been observed with around 70% of physicians followed ESHRE guidelines [3].Consistent with global patterns, the Rotterdam/ESHRE criteria, introduced in 2003, were widely utilized, requiring two of three key features for diagnosis: irregular or absent ovulation, signs of hyperandrogenism, and polycystic ovaries on ultrasound, excluding other conditions with similar symptoms. As PCOS presents in many different ways, the NIH workshop stressed the importance of identifying and studying specific PCOS types to better understand their impact, severity, and long-term effects across different patient groups [7,21].

The study ensured balanced geographic representation, with nearly equal participation from physicians practicing outside Dhaka. Over half of the respondents had more than a decade of clinical experience, reflecting a high level of expertise. The predominance of gynecologists and obstetricians aligns with the study’s reproductive health focus, corroborated by earlier research that identified gynecologists as the preferred specialists for referral [22]. Diagnostic practices frequently included ultrasound and hormonal assays, reflecting global trends. However, a notable emphasis on thyroid function tests, such as serum TSH, was observed, diverging from Western practices that prioritize metabolic markers and insulin resistance [23].

Physicians in the study managed approximately 500 patients per week, including 20 PCOS cases, underscoring the condition’s prevalence. In the United States and Europe, lifestyle modifications, particularly weight loss, are considered the first-line treatment for PCOS, with metformin and oral contraceptives commonly prescribed in these regions [14,24]. Aerobic exercise, in particular, has been shown to improve reproductive function in women with PCOS by normalizing menstrual cyclicity and ovulation rates. Improvements in insulin sensitivity through exercise are associated with increased menstrual regularity and ovulation in approximately 50% of women with PCOS [9]. Research suggests that lifestyle interventions, such as aerobic exercise, improve reproductive outcomes by normalizing menstrual cycles and enhancing insulin sensitivity. Metformin, commonly prescribed for PCOS-related infertility, is administered following standard protocols of gradual dose titration [12,25,26].

Most physicians schedule follow-ups every four to 12 weeks, using tests such as serum TSH levels and abdominal ultrasound to monitor treatment efficacy and complications. This follow-up frequency aligns with global recommendations, emphasizing the importance of long-term management and screening for diabetes and cardiovascular disease [27,28]. The primary long-term concerns for PCOS patients include infertility, obesity, type 2 diabetes, psychological issues, endometrial cancer, and cardiovascular disease. Suggestions for improving PCOS care included educational materials, webinars, and professional development resources, underscoring the need for enhanced support for healthcare providers [29].

Moreover, this study assessed some important factors influencing physician adherence to PCOS guidelines. Physicians practicing within Dhaka were significantly more likely to adhere to guideline-based management compared to those outside Dhaka. This may reflect better access to continuing medical education, academic networks, and exposure to updated clinical resources in urban centers, which are often limited in peripheral regions. Similar urban-rural disparities were found in other studies on various topics, underscoring the need to strengthen training opportunities and resource availability in rural settings here [30,31]. Interestingly, physicians who preferred referring PCOS patients to specialists had lower odds of adhering to guidelines. This could indicate limited confidence in managing PCOS cases themselves or an overreliance on referral pathways. While referral is appropriate for complex cases, excessive referral may delay the timely initiation of evidence-based care. Interventions aimed at empowering non-specialist physicians through targeted training and decision-support tools could enhance guideline adherence in such contexts.

PCOS remains a prevalent global health issue, presenting reproductive, metabolic, and psychological challenges. Early diagnosis and consistent management are vital for improving patient outcomes. This study, one of the largest in Bangladesh, offers a comprehensive analysis of nationwide physician practices and highlights opportunities to address gaps in knowledge and guideline adherence. The findings emphasize the need for educational initiatives aligned with evidence-based guidelines and suggest that future research should focus on patient-centered care models to enhance satisfaction and outcomes.

This study has several limitations. The predominance of gynecologists among respondents may have introduced sampling bias, emphasizing reproductive aspects over metabolic or psychological dimensions. Reliance on self-reported practices and the absence of patient outcome data further limit the validity of the findings. The exclusion of patient perspectives restricts understanding of care experiences and satisfaction. Moreover, the use of convenience sampling means that results may not be generalizable beyond the study respondents or to other healthcare settings. Future studies should adopt mixed-methods approaches incorporating both physician and patient perspectives to better capture practice patterns, care gaps, and contextual barriers. At the policy level, promoting adherence to clinical guidelines through targeted training, structured referral systems, and wider dissemination of international recommendations alongside emphasis on lifestyle interventions, potentially supported by mindfulness techniques, may help improve patient outcomes.

## Conclusions

In summary, this nationwide survey offers valuable insights into current PCOS management practices among Bangladeshi physicians, particularly regarding their use of established international guidelines. Most respondents reported familiarity with at least one evidence-based recommendation; however, uniform adherence to a single guideline tailored to the Bangladeshi context is lacking. Variability in follow-up and referral practices further highlights the urgent need for structured referral pathways and a standardized care model for PCOS diagnosis and management among physicians. Moreover, implementing targeted training modules, strengthening the dissemination of international guidelines, and introducing system-level interventions could enhance practice consistency and ultimately improve patient outcomes. Beyond its national relevance, this study makes an important global contribution by offering rare insights from a South Asian LMIC (low- and middle-income countries) context, which may enable scopes of future cross-country comparisons on physician practices and inform strategies to improve consistency of PCOS care in resource-constrained settings.

## Appendices

Survey

Please Tick (√) the Suitable Option/s

(You can choose multiple options for a single question)

Sl.

Question

Please tick (√) the suitable option/s (You can choose multiple options for a single question)

1.

What is your current area of specialty?

a) Endocrinology

b) Internal Medicine

c) Gynecology & Obstetrics

d) Cardiology

e) Nephrology

f) Psychiatry

g) Dermatology

h) Surgery

i) Others (Kindly specify) ________________________

2.

Years of experience in clinical practice-

a. <5 years

b. 6-10 years

c. 10-15 years

d. >15 years

3.

What is your current area of practice?

(Kindly specify) __________________________________

4.

How many patients do you get during your clinical practice?

Daily _________________

Weekly __________________

5.

How many patients with polycystic ovary syndrome (PCOS) do you get during your clinical practice in last month?

O Less than 5

O 5-20

O More than 20

6.

What is your opinion about PCOS awareness among your patients?

a. Poor

b. Average

c. Good

d. Excellent

7.

Which guidelines do you usually follow to screen/diagnose PCOS disorders?

O National Institutes of Health (NIH)

O Rotterdam/European Society for Human Reproduction and Embryology (ESHRE)

O Androgen Excess and PCOS Society

O . Evidence Based Guideline

O Don’t know

O If other, please specify

___________________________________

8.

Generally according to which guideline do you manage your patients with PCOS?

O National Institutes of Health (NIH)

O Rotterdam/European Society for Human Reproduction and Embryology (ESHRE)

O Androgen Excess and PCOS Society

OEvidence Based Guideline

O Don’t know

O If other, please specify

___________________________________

9.

Which clinical feature do you encounter in clinical practice dealing with PCOS?

(please select as many as you feel appropriate, this is a general list and not all may have been associated with PCOS)

Irregular menstrual cycles/periods

Premenstrual syndrome (PMS)

Migraines

Increased tendency for weight gain

Difficulty losing weight

Improvement of symptoms with a low glycemic index (GI) diet

Improvement of symptoms after weight loss

Improvement of symptoms with exercise

Reduced quality of life

Depression

Anxiety

Body image dissatisfaction

Hormone imbalance

Insulin resistance

Increased risk of type 2 diabetes

Increased risk of cardiovascular disease risk factors

High blood androgen levels

Excess hair growth

Scalp hair loss/male pattern-baldness

Acne

Ovarian cancer

Endometrial cancer

Surgery for ovarian cysts

Reduced fertility

Infertility

Cysts on ovaries

Pregnancy complications

Increased risk of gestational diabetes

Fatty liver

Sleep apnea and snoring

10.

Of those diagnosed with PCOS, what was the most common reason for clinic attendance?

(Please select the most common reasons)

Hirsutism

Infertility

Menstrual disturbances

Metabolic disorders

Obesity

11.

For suspected PCOS which are the tests you usually suggest?

a. Serum FSH

b. Serum LH

c. TSH

d. Prolactin

e. Total testosterone

f. DHEA

g. Free testosterone

h. Fasting insulin

i. 17(OH) progesterone

j. OGTT

k. Fasting lipid profile

l. AMH

m. USG of the abdomen

n. Serum cortisol

o. Others (Kindly specify) ____________________________________________

12.

For children aged less than 18 years with suspected PCOS which are the tests you usually suggest?

a. Serum FSH

b. Serum LH

c. TSH

d. Total testosterone

e. DHEA

f……….. Free testosterone

g……… 17(OH) progesterone

h………. Prolactin

k. Others (Kindly specify)

________________________________

13.

Which test do you prefer for following up of PCOS patients?

a. Serum FSH

b. Serum LH

c. TSH

d. Total testosterone

e. DHEA

f. USG of the abdomen

g……….. Electrolyte

h………… Blood sugar

k. Others (Kindly specify)

_______________________________

14.

Do you think polycystic or multiple cysts in ovaries is necessary for diagnosis of PCOS?

a. Yes

b. No

c. Sometime

d. Don’t know

15.

How frequently do you follow up patients with PCOS?

a. In every 6 weeks

b. In every 12 weeks

c. In every 6 months

d. In every 12 months

e. More frequently (Kindly specify) __________________________________

f. Never (Please specify the reason) ____________________________________

16.

What is your preferred drug as a starting of management of PCOS?

a. Anti-androgens

b. Progesterone only

c. Laser depilation

d. Lifestyle modifications

e. Metformin

f. Oral contraceptives

g. Others (Please write the name of the drug(s)______________________________________

17.

What is your preferred dose of prescribed drug as a starting of management of PCOS?

____________________________________(Write the name of the drug first and write the dose thereafter)

18.

What is your common approach to a patient with confirmed PCOS?

a. Follow up

b. Optimising treatment

c. Counselling of the patient

d. Referred to respective specialist

19.

Do you feel a patient with PCOS come for follow-up visit as suggested?

a. Yes

b. No

20.

How frequently do you refer a patient with PCOS to an Endocrinologist/Gynecologist/Medicine/Dermatologist/Others?

a. Never

b. Rarely

c. Frequently

d. Very frequently

e. All cases

21.

What is your most important long-term concern about PCOS?

O Infertility

O Cardiovascular diseases

O Obesity and type 2 diabetes

O Endometrial cancer

O Psychosocial problems

O If other, please specify

___________________________________

22.

How can clinicians and health professionals be best supported in caring for women with PCOS?

(Please select as many as preferred)

Provide broadly available educational materials for health professionals

Presentations at health professional forums and workshops

Maintain a health professional website on PCOS

Send a regular health professional update email on PCOS

Provide resources for patients e.g. education materials, presentations at patient forums, maintain a consumer website

Other

___________________________________

23.

In general, do you think that women with PCOS are correctly diagnosed as such in your country of practice?

O Yes, I think that PCOS is correctly diagnosed in the majority of cases and correctly excluded when a woman does not have PCOS

O No, I think PCOS is under-diagnosed

O No, I think PCOS is over-diagnosed

O I don’t know

------- Thank you for your sincere contribution to the survey --------

## References

[1] Yasmin A, Roychoudhury S, Paul Choudhury A (2022). Polycystic ovary syndrome: An updated overview foregrounding impacts of ethnicities and geographic variations. Life (Basel).

[2] Singh S, Pal N, Shubham S, Sarma DK, Verma V, Marotta F, Kumar M (2023). Polycystic ovary syndrome: Etiology, current management, and future therapeutics. J Clin Med.

[3] Dokras A, Saini S, Gibson-Helm M, Schulkin J, Cooney L, Teede H (2017). Gaps in knowledge among physicians regarding diagnostic criteria and management of polycystic ovary syndrome. Fertil Steril.

[4] Vidya Bharathi R, Swetha S, Neerajaa J (2017). An epidemiological survey: Effect of predisposing factors for PCOS in Indian urban and rural population. Middle East Fertil Soc J.

[5] Kamrul-Hasan AM, Aalpona FZ, Mustari M, Selim S (2023). Prevalence and characteristics of women with polycystic ovary syndrome in Bangladesh - A narrative review. Bangladesh J Endocrinol Metab.

[6] Azziz R, Carmina E, Dewailly D (2009). The Androgen Excess and PCOS Society criteria for the polycystic ovary syndrome: The complete task force report. Fertil Steril.

[7] Rotterdam ESHRE/ASRM-Sponsored PCOS Consensus Workshop Group (2004). Revised 2003 consensus on diagnostic criteria and long-term health risks related to polycystic ovary syndrome (PCOS). Hum Reprod.

[8] Kaundal A, Renjhen P, Kumari R (2023). Awareness of lifestyle modifications in the management of polycystic ovarian syndrome: A hospital-based descriptive cross-sectional study. Cureus.

[9] Barber TM, Hanson P, Weickert MO, Franks S (2019). Obesity and polycystic ovary syndrome: Implications for pathogenesis and novel management strategies. Clin Med Insights Reprod Health.

[10] Lee IT, Sansone S, Irfan M, Copp T, Beidas R, Dokras A (2022). Implementation of international guidelines for polycystic ovary syndrome: Barriers and facilitators among gynecologists and primary care providers. F S Rep.

[11] Teede HJ, Misso ML, Costello MF (2018). Recommendations from the international evidence-based guideline for the assessment and management of polycystic ovary syndrome. Fertil Steril.

[12] Gibson-Helm M, Dokras A, Karro H, Piltonen T, Teede HJ (2018). Knowledge and practices regarding polycystic ovary syndrome among physicians in Europe, North America, and internationally: An online questionnaire-based study. Semin Reprod Med.

[13] Chemerinski A, Cooney L, Shah D, Butts S, Gibson-Helm M, Dokras A (2020). Knowledge of PCOS in physicians-in-training: Identifying gaps and educational opportunities. Gynecol Endocrinol.

[14] Piltonen TT, Ruokojärvi M, Karro H (2019). Awareness of polycystic ovary syndrome among obstetrician-gynecologists and endocrinologists in Northern Europe. PLoS One.

[15] Ma R, Zou Y, Wang W (2021). Obesity management in polycystic ovary syndrome: Disparity in knowledge between obstetrician-gynecologists and reproductive endocrinologists in China. BMC Endocr Disord.

[16] Mahjabeen N, Nasreen SZA, Mustary F (2020). Clinical profile of 500 cases of polycystic ovary syndrome in a tertiary hospital. Bangladesh J Obs Gynaecol.

[17] Khan M, Jaman A, Ahmed MF, Das N (2024). Understanding polycystic ovary syndrome (PCOS) and mental health disparities in Bangladeshi women: A mixed method approach [preprint]. Research Square.

[18] Hasan MJ, Rafi MA, Nishat NH (2024). Patient self-referral patterns in a developing country: Characteristics, prevalence, and predictors. BMC Health Serv Res.

[19] Hasan MJ, Hossain MZ, Hossain MA (2024). Health-care-seeking behaviour in patients with hypertension: Experience from a dedicated hypertension centre in Bangladesh. Blood Press.

[20] Naz MS, Tehrani FR, Majd HA, Ahmadi F, Ozgoli G, Fakari FR, Ghasemi V (2019). The prevalence of polycystic ovary syndrome in adolescents: A systematic review and meta-analysis. Int J Reprod Biomed.

[21] Bozdag G, Mumusoglu S, Zengin D, Karabulut E, Yildiz BO (2016). The prevalence and phenotypic features of polycystic ovary syndrome: A systematic review and meta-analysis. Hum Reprod.

[22] Sydora BC, Wilke MS, McPherson M, Chambers S, Ghosh M, Vine DF (2023). Challenges in diagnosis and health care in polycystic ovary syndrome in Canada: A patient view to improve health care. BMC Womens Health.

[23] Spira D, Buchmann N, Dörr M (2022). Association of thyroid function with insulin resistance: Data from two population-based studies. Eur Thyroid J.

[24] Cowan S, Lim S, Alycia C (2023). Lifestyle management in polycystic ovary syndrome - Beyond diet and physical activity. BMC Endocr Disord.

[25] Gu Y, Zhou G, Zhou F (2022). Life modifications and PCOS: Old story but new tales. Front Endocrinol (Lausanne).

[26] Angel C, Packer CD (2019). Analysis of medical error contributing to missed acute myeloid leukemia diagnosis. Cureus.

[27] Lobo RA, Carmina E (2000). The importance of diagnosing the polycystic ovary syndrome. Ann Intern Med.

[28] Hudecova M, Holte J, Olovsson M, Sundström Poromaa I (2009). Long-term follow-up of patients with polycystic ovary syndrome: Reproductive outcome and ovarian reserve. Hum Reprod.

[29] Palomba S, Santagni S, Falbo A, La Sala GB (2015). Complications and challenges associated with polycystic ovary syndrome: Current perspectives. Int J Womens Health.

[30] Khoong EC, Gibbert WS, Garbutt JM, Sumner W, Brownson RC (2014). Rural, suburban, and urban differences in factors that impact physician adherence to clinical preventive service guidelines. J Rural Health.

[31] Cyr ME, Etchin AG, Guthrie BJ, Benneyan JC (2019). Access to specialty healthcare in urban versus rural US populations: A systematic literature review. BMC Health Serv Res.

